# Supplementary material for: Neonatal Drug Formularies—A Global Scope
Source: Children (Basel). 2023 May 8;10(5):848. doi: 10.3390/children10050848 (PMC10217712; doi:10.3390/children10050848)
Supplement: Supplementary file 1 [file children-10-00848-s001.zip › Document S1 - Questionnaire for the Formularies ver. 3.2.pdf]

Questionnaire for the formularies, version 3.2, 19.10.2022

When was the formulary first published?

1. What format(s) is it published in?
    a. Web-based
    b. Mobile application
    c. Print
2. Who is the target audience?
3. What language(s) is it published in?
4. Is it linked to a broader formulary (adults, children) or is it a standalone formulary?
5. What is the business model?
    a. Individual subscription
    b. Institutional subscription
    c. Embedded in other applications
    d. Free to some?
6. What is the frequency of updates?
    a. Are there “major” updates and “minor” updates?
    b. Are updates always scheduled, or does new evidence lead to an immediate update?
7. How are updates communicated to users?
8. How are drugs selected for inclusion?
    a. How are drugs first identified for possible inclusion?
    b. How is it determined when there is enough need and/or evidence for inclusion?
9. Who is involved in the development of a monograph?
    a. Expertise
    b. Hierarchy of decision making
10. What is the search process for literature support?
    a. Are specific Pub Med terms used on all drugs?
    b. Is there a “deeper dive” into references listed in the initial articles retrieved?
    c. What is considered “best-evidence”?
    d. How are expert panel recommendations utilized?
11. Is the dosing information evaluated differently than other sections of the monograph?
    a. If there is not a single standard dose, are multiple doses mentioned versus a range?
12. Are extrapolations done from PK / PD data in children?
13. How is product labeling used in constructing the drug monograph?
    a. What if the labeling does not mention neonates?
    b. What if the labeling has a vague warning about use under a certain age?
    c. How are black box warnings included in the monograph?

Any free text that the formulary ‘holders’ feel relevant to share
