# Supplementary material for: Neonatal Drug Formularies—A Global Scope
Source: Children (Basel). 2023 May 8;10(5):848. doi: 10.3390/children10050848 (PMC10217712; doi:10.3390/children10050848)
Supplement: Supplementary file 1 [file children-10-00848-s001.zip › Document S2 - Data Extraction Tool.pdf]

Parameters

- Is a drug monograph available?
- Dosage based on which parameter (weight/age/route of administration)?
- Is method of administration indicated?
- Number of routes of administration
- Number of age groups
- Number of indications (including general dosage)
- Differentiation between preterm and term patients
- Are monitoring advice and parameters indicated (if applicable)?
- Renal dosage adjustment (if applicable)
- Hepatic dosage adjustment (if applicable)
- Other dosage adjustments (if applicable)
- Drug-drug interactions mentioned?
- Adverse effects mentioned?
- If adverse effects are mentioned, are age-specific effects mentioned?
- Are preparation instructions available?
- Are stability data provided (if applicable)?
- Optimal concentration of preparation (if applicable)
- Maximum concentration of preparation (if applicable)
- Are Notable excipients mentioned?
- Are commercial products listed?
- Are pharmacokinetic data available?
