# Supplementary material for: Neonatal Drug Formularies—A Global Scope
Source: Children (Basel). 2023 May 8;10(5):848. doi: 10.3390/children10050848 (PMC10217712; doi:10.3390/children10050848)
Supplement: Supplementary file 1 [file children-10-00848-s001.zip › Document S3 - Summary of Key Aspects of Studied Formularies.pdf]

This supplementary document aims to complement the brief description of the studied neonatal formularies in the main text and shed more light on certain aspects of their workflow, method of publication and update, and monograph presentation.

3.2.1. Australasian Neonatal Medicines Formulary (ANMF)

Workforce and -flow: The ANMF team is comprised of a steering group and reference groups: the steering group is responsible for successful running of the project using a step-wise process of (1) identification of medications requiring a consensus formulary, (2) development of an evidence-based yet pragmatic paper-based and electronic formulary pertinent to Australasian region, (3) collaboration with all 29 tertiary NICUs across Australia and New Zealand and many special nurseries across Australia and New Zealand to achieve a majority consensus, (4) implementation of formulary in the clinical area, (5) feedback and evaluation within 3 months of implementation across Australia and New Zealand and revision as required, based on feedback, (6) regular review of formularies. The primary author is one of the steering group members of the ANMF, often a neonatologist but occasionally a steering group nurse or pharmacist will write the initial draft. Reference groups rigorously discuss the monograph in the weekly meetings, verify the accuracy and interpretation of the studies referenced in the draft and debate the applicability of the available evidence to the bedside practice. In addition, the Chair of the ANMF chooses an expert to contact for each monograph, as required. Thus, each monograph may take 6-8 weeks to complete. Consensus must be reached before content is approved and published.

ANMF protocols follow the National Health and Medical Research Council (NHMRC) hierarchy of levels of evidence and grades for recommendations guide wherever applicable[1].

Publication and update: Published protocols are available online in English in a searchable library within the ANMF website (www.anmfonline.org). Content is reviewed periodically (1 to 5 years) according to ANMF review process, with current and next review date stated at the bottom of each monograph.

Monograph presentation: ANMF monographs are provided as PDF files under the 'Clinical Resources' section of the ANMF website. This section lists all available monographs in two groups – Current Monographs and New Monograph Releases, both searchable. The monographs have a standard structure, which includes version numbers and dates, scheduled date for review and a list of contributing authors. References are provided in-line, i.e., directly next to most statements, and are also collectively listed in the end of the monograph. One feature that stands out is that each monograph also provides an elaborate section titled 'Evidence', which summarizes the literature on important pharmacological aspects of the drug, including but not limited to efficacy, safety, pharmacokinetics, and more.

3.2.2. British National Formulary for Children (BNF for Children)

Workforce and -flow: Content in BNF for Children is produced by an editorial team, with oversight provided by a Pediatric Formulary Committee (PFC), comprised of pharmacists, doctors, nurses and other

healthcare professionals with pediatric background, as well as laypersons, and representatives of government departments, such as the Medicines and Healthcare products Regulatory Agency (MHRA) and the UK Departments of Health. The work of the PFC is complemented by the Dental Advisory Group, which is responsible for information around dental and oral conditions, the Nurse Prescribers' Advisory Group, responsible for information in the Nurse Prescribers' Formulary, and a network of expert advisers from professional societies and advisory bodies.

The editorial team is comprised of experienced pharmacists, some with specific experience in pediatric practice. The team integrates information from a range of sources, including SmPCs, primary literature, consensus guidelines, reference sources (e.g., Martindale: The Complete Drug Reference), government and statutory information, NHS Prescription Services, and expert advice. Recommendations in BNF for Children are evidence-graded according to a five-level grading system from A to E, while evidence is graded by numerical levels from level 1++ (high quality meta-analyses, systematic reviews of randomized controlled trials, etc.) to level 4 (expert advice or clinical experience from respected authorities). Recommendations whose evidence base is weak or contradictory are assessed directly by the PFC and some are additionally subject to peer review before publication. DI is reviewed and updated both in response to new evidence from surveyed literature and proactively, with the aim to consider all recommendations for review every 3-4 years.

Publication and update: the print version of BNF for Children is published – and therefore updated – annually in September; the digital version of BNF for Children is updated monthly in accordance with observed updates to the relevant medical literature. In addition, the most clinically significant updates are collected and listed in a dedicated section of the content.

Monograph presentation (in MedicinesComplete®): BNF for Children monographs are uniformly structured and are divided into sections, and a sidebar is available for easy navigation. Notable monograph sections include (as appropriate): drug action, indications and dose (where neonatal dosing recommendations are specifically indicated, if available), unlicensed use, important safety information, cautions, interactions, side-effects, conception and contraception, pregnancy, breastfeeding, pre-treatment screening, monitoring requirements, directions for administration, prescribing and dispensing information, medicinal forms and other drugs in class. Moreover, each monograph has a digital object identifier (DOI) and the date of last update at the top of the monograph, and includes a link to the SmPC of the manufacturer from the Electronic Medicines Compendium (EMC) of the UK. No specific references are cited in the monograph.

3.2.3. The 'Dutch' Pediatric Formulary (DPF, also known as 'Kinderformularium')

Workforce and -flow: Drugs that are licensed for use in children are included in the formulary by default. If a product is licensed for use in a certain age group, compliance with the SmPC is preferred unless there are strong arguments to deviate from it. If a European SmPC does not explicitly mention use in neonates, DPF considers the drug off-label for use in this age group. Drugs that are not authorized for the pediatric

population (off-label use) are included in DPF if deemed necessary by pediatric professionals. For off-label use, a drug monograph is developed by a senior pharmacist using a structured framework, by assessing available scientific evidence that support dose recommendations, efficacy and related safety issues. No minimum level of evidence is required, but information regarding experimental use is not included. Available evidence is summarized in dose rationale (benefit-risk) documents and presented for peer-review to a multidisciplinary editorial board consisting of pediatricians with diverse specialties, clinical pharmacologists, pharmacists, pediatric hospital pharmacists and primary care physicians. Special expert groups are available for neonatology, nephrology and pharmacokinetic model informed dosing, and if deemed necessary, other experts may be consulted. The drugs monographs are published online in the respective DPF platforms of the Netherlands, Germany, Austria and Norway, including citations and references. The full dose rationale summaries are not published, but are available upon request.

A dedicated project to further develop the neonatal dosing regimens (NEODOSE) has recently been reported[13].

Publication and update: Monographs require unanimous approval by the editorial board prior to publication. Following publication, they are updated every 5 years, or more frequently as new evidence emerges. Changes are indicated within the monographs and are also communicated to users in the form of monthly updates and through learned societies.

Monograph presentation: DPF Monographs are comprised of "best-evidence", which is scientific evidence on PK, efficacy and safety of the drug, complemented with clinical expertise and extrapolation (preferably using published PK data) where published evidence is limited, as is common in neonatal clinical pharmacology. Monograph content includes common data sections such as drug properties (PK and PD), licensing information, dosing, available formulations including excipients information, dosing in renal impairment (applicable above 3 months of age), side effects, warnings and precautions, contraindications, drug interactions, references and monograph history of changes.

3.2.4. NeoFax

Workforce and -flow: Monographs are created or updated through weekly surveillance of published neonatal literature and are written and peer-reviewed by internal pharmacist editors, while a final review for new neonatal monographs and new off-label indications is done by an external peer reviewer with neonatal/pediatric expertise. The editorial team also tracks U.S. regulatory product information. If this contains neonatal-specific information, it will be incorporated into the monograph where appropriate. Where dosing recommendations from product information and published literature differ, both will usually be included in the monograph. Extrapolation from pediatric studies is not implemented unless it has been published in the available literature.

Publication and update: NeoFax is updated and published monthly, unless new safety or practice-changing information becomes available, in which case it is updated outside of the regular publishing schedule. Updates are communicated to users via a dedicated section titled 'What's

New', which lists new drug monographs, updated monographs, and drugs with dosage recommendation changes.

Monograph presentation: Monographs in NeoFax are divided into sections and subsections as follows (section titles appear between single quotations marks, subsections in parentheses): 'Dosing/Administration' (Dose, Uses, Administration), 'Medication Safety' (Contraindications/Precautions, Adverse Effects, Black Box Warning, Solution/Drug Compatibility, Monitoring), 'Mechanism of Action/Pharmacokinetics' and 'About' (Special Considerations/Preparation, References). Monographs do not specify time of latest update or a scheduled time for review. References are provided next to most statements, and are also listed together in a separate subsection.

3.2.5. Neonatal Formulary (NNF)

Workforce and -flow: The data provided in NNF rely on information collected from UK approved SPCs and published literature (including Cochrane reviews where appropriate), and, where no substantial data can be found, on professional expertise of local and overseas collaborators. Special attention is given to renal and hepatic function and their effects on drug exposure, toxicity due to overdosing and the effects of non-pharmacologic treatments such as therapeutic hypothermia (TH) on drug PK, where applicable.

Monograph presentation: NNF is divided into 3 parts: part 1 contains general information about clinical neonatal pharmacotherapy, e.g., adverse reactions, PK/PD, dosing, administration, storage, legal aspects (licensing and prescribing), excipients. These sections provide summaries of important and unique concepts for NICU settings. Part 2 contains individual drug monographs for drugs commonly used in NICUs, in which specific DI is provided in specific subsections, e.g., 'Use', 'Pharmacology', 'Fetal and infant implications of maternal treatment' and 'Supply'. Additional subsections are present as applicable, such as antidotes, effects of TH, or therapeutic drug monitoring (TDM), making NNF monographs non-uniform in structure. Certain monographs have additional online-only content available at the website of the publisher (see Table 1). The availability of such content is clearly marked at the top of the monograph, along with the title of the supplementary material. Part 3 is dedicated to exposure to maternal medications *in utero*, during labor or in the postpartum period, and provides information on specific aspects of drug safety of maternal medications such as placental transfer, teratogenicity and drug excretion into breastmilk. The information about each drug in this section is concise and abridged, but key references are given for the reader.

Publication and update: the printed version of NNF is being updated every couple of years and a new edition is published and made available for purchase. Online supplementary material is updated whenever significant changes in dosing occur.

3.2.6. Pediatric and Neonatal Lexi-Drugs (Lexicomp)

Workforce and -flow: Pediatric and Neonatal Lexi-Drugs employs a multi-step, peer-reviewed editorial process by in-house pediatric/neonatal residency trained clinical pharmacists and an external

neonatal advisory panel, which includes neonatologists and neonatal clinical pharmacists (a detailed list is available online[14]). Pubmed searches are conducted according to specific terms and keywords, and results are evaluated according to an adapted grading system for design, methodology, statistical analysis, results and overall quality of the paper before it is deemed appropriate for inclusion in the drug monograph. Ultimately, best evidence is synthesized from peer-reviewed literature, published clinical guidelines, expert opinions of neonatal practitioners, and any applicable manufacturer labeling. No extrapolation of older children/adult PK data is employed in the formulation of drug dosing recommendations. If available data in a certain aspect are vague or limited, this is clearly indicated adjacently to those data.

Monograph presentation: neonatal DI is provided as part of the Pediatric and Neonatal Lexi-Drugs drug monograph. Drug monographs in Lexicomp are uniformly structured, and include a sidebar containing an outline of the monograph for easy navigation and a main view presenting the various aspects of DI, including dosing recommendations according to indications, preparation and administration instructions, contraindications, adverse events, warnings/precautions, monitoring information, pharmacokinetics (usually adult data unless otherwise noted) and many other fields – all indicate neonatal DI where available. Citations appear in-line and are also grouped together in the references section of the monograph. The PubMed ID (PMID) number is included and leads to the appropriate Pubmed abstract, with external links as well. Boxed warnings (also known as "black box" warnings) are included in the Warnings section per the wording in the U.S. manufacturer labeling.

Publication and update: new content is released online on a daily basis and is available to the user immediately. As new evidence becomes available, it is processed and prioritized based on its importance to healthcare providers. Major updates are implemented immediately (e.g., practice-changing evidence, new medications), while minor updates are published in a timely manner as possible. Updates to users are made through monthly newsletters sent by the marketing department or may be sent by the parent formulary Lexicomp is part of at a specific institution.

References

1. NHMRC additional levels of evidence and grades for recommendations for developers of guidelines – Stage 2 Consultation. Available online: https://www.mja.com.au/sites/default/files/NHMRC.levels.of.evidence.2008-09.pdf (accessed on 14 December 2022)
2. de Hoop-Sommen, M. A., van der Zanden, T. M., Allegaert, K., Flint, R. B., Simons, S. H. P., de Wildt, S. N., & Working group Neonatal Pharmacology (NeoDose project) (2023). Development of Best Evidence Dosing Recommendations for Term and Preterm Neonates (NeoDose Project). Neonatology, 1–12. Advance online publication. https://doi.org/10.1159/000528012
3. Neonatal Editorial Advisory Panel (Pediatric and Neonatal Lexi-Drugs). Available online: https://online.lexi.com/lco/action/doc/retrieve/docid/pdh_f/3474962 (accessed on 28 March 2023)
